# Structure of Organoboron Dyes and Multiphoton Absorption: Insights from Theory

**DOI:** 10.1021/acs.jpclett.5c02066

**Published:** 2025-09-09

**Authors:** Karan Ahmadzadeh, Natasza Trzęsowska, Rafał Wysokiński, Zilvinas Rinkevicius, Robert Zaleśny, Wei Hu, Borys Ośmiałowski, Hans Ågren

**Affiliations:** † Hefei National Research Center for Physical Sciences at the Microscale, 12652 University of Science and Technology of China, Hefei, Anhui 230026, China; ‡ Faculty of Chemistry, 49567 Wrocław University of Science and Technology, Wyb. Wyspiańskiego 27, PL-50370 Wrocław, Poland; ¶ Division of Theoretical Chemistry and Biology, School of Engineering Sciences in Chemistry, Biotechnology and Health, 7655 KTH Royal Institute of Technology, SE-100 44 Stockholm, Sweden; § Faculty of Chemistry, Nicolaus Copernicus University, Gagarina Street 7, Toruń PL-87-100, Poland

## Abstract

Computer simulations play an essential role in the interpretation of experimental multiphoton absorption spectra. In addition, models derived from theory allow for the establishment of “structure-property” relationships. This work contributes to these efforts and presents the results of an analysis of two- and three-photon absorptions for a set comprising 450 conjugated molecules performed at the CAM-B3LYP/aug-cc-pVDZ level. The molecular set is composed of organoboron dyes presenting various core topologies combined with a palette of conjugated linkers giving donor–acceptor architectures. The charge-transfer character of the investigated structures is manifested by the presence of the low-lying electronic excited state. The multiphoton excitation to the state in question is intense and significant from an application point of view. The analysis performed in this work clearly demonstrates that there is a strong correlation between the intensities of the two- and three-photon transitions to the lowest intramolecular charge-transfer state, hinting that developed design rules aiming at maximizing two-photon absorption efficiency will also be useful in designing three-photon absorbers. As part of this study, we also performed two-photon absorption calculations using the coupled-cluster RI-CC2 model with the aug-cc-pVDZ basis set for 450 molecules to guide the selection of the density functional approximation.

## Graphical abstract

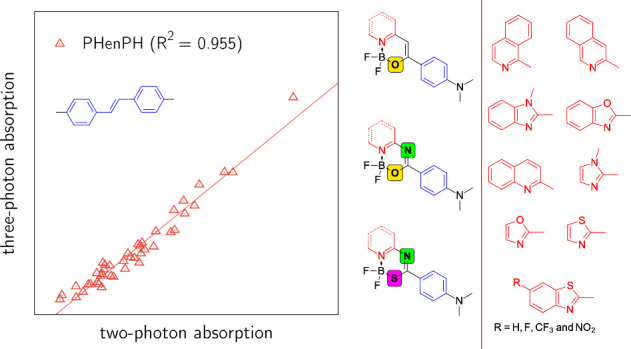

Thorough understanding of light-matter interactions in the strong-field regime is pivotal for the design of nonlinear optical materials with tailored properties. An example of nonlinear optical phenomena with significant application potential is multiphoton absorption. The simultaneous absorption of two, three,^1–3^ or more^2,4^ photons enables transition from the ground state to a higher excited state, provided a resonance condition is fulfilled. The most well-studied multiphoton absorption process is two-photon absorption (2PA).^5^ 2PA spectroscopy can be used in studies of electronic structure of molecules,^6,7^ especially to probe the states that are dark with usual one-photon absorption (1PA) spectroscopies. Transitions to dark states might be forbidden due to selection rules or simply too weak to be detected in 1PA measurements. There are also numerous reports of applications of two-photon absorption in photodynamic therapy,^8–10^ microfabrication,^11,12^ material sciences,^5,13^ data storage,^14,15^ and bioimaging.^16–20^ In the case of the latter area, by employing two-photon-excited fluorescence probes, it is possible to reduce scattering effects, enhance resolution, and increase penetration depths. These developments were paralleled by more fundamental studies aiming at establishing structure–property relationships for two-photon absorbers.^5,21–24^ Despite these efforts^1^ and theoretical developments made,^25^ three-photon absorption (3PA) in molecular systems has been less extensively studied even though this process demonstrates significant application potential, e.g., in three-photon fluorescence microscopy which enables imaging deeply in tissues.^26–30^

It is not yet established to what extent the chemical design rules of two-photon absorbers are transferable to the three-photon absorption, i.e., whether the same parameters maximize the efficiency. A recent study by Chołuj et al.,^31^ who employed generalized few-state models to link two- and three-photon absorption activity with electronic structure parameters, suggests that for dipolar donor–acceptor π-conjugated chromophores the mechanism of these two processes is similar; i.e., 2PA and 3PA activity is governed by the final intramolecular charge-transfer state (transition moment between the ground electronic state and final state, dipole moment in the final state). The study in question focused only on three Y-shaped π-conjugated molecules, and this interesting observation requires further thorough studies for dipolar compounds that usually are easier to synthesize. In particular, the development of three-photon absorbers as molecular probes for microscopy requires fulfilling additional requirements, e.g., tuning of absorption wavelengths, 3PA transition strengths, and fluorescence quantum yields. The goal of the present Letter is to contribute to development of design rules for three-photon absorbers by studying a wide palette of boron-carrying, charge-transfer dyes. In more detail, the systems investigated in this study, shown in Scheme 1, are based on organoboron chromophores, where a central acceptor core is functionalized with a palette of moieties of varying structural complexity. Our focus on this family stems from the fact that organoboron fluorescent dyes are studied extensively as they exhibit significant application potential.^32–44^ This interest also follows from the excellent photophysical properties of organoboron dyes with BF/BF_2_ groups, which can be tuned by structural modifications. Functionalization of these dyes might influence the absorption/emission band positions^45,46^ as well as fluorescence quantum yields.^47–51^ Provided that the absorption wavelength fits into the desired spectral region, difluoroborates can be used in multiphoton microscopy due to their significant fluorescent quantum yields.

**Scheme 1:**
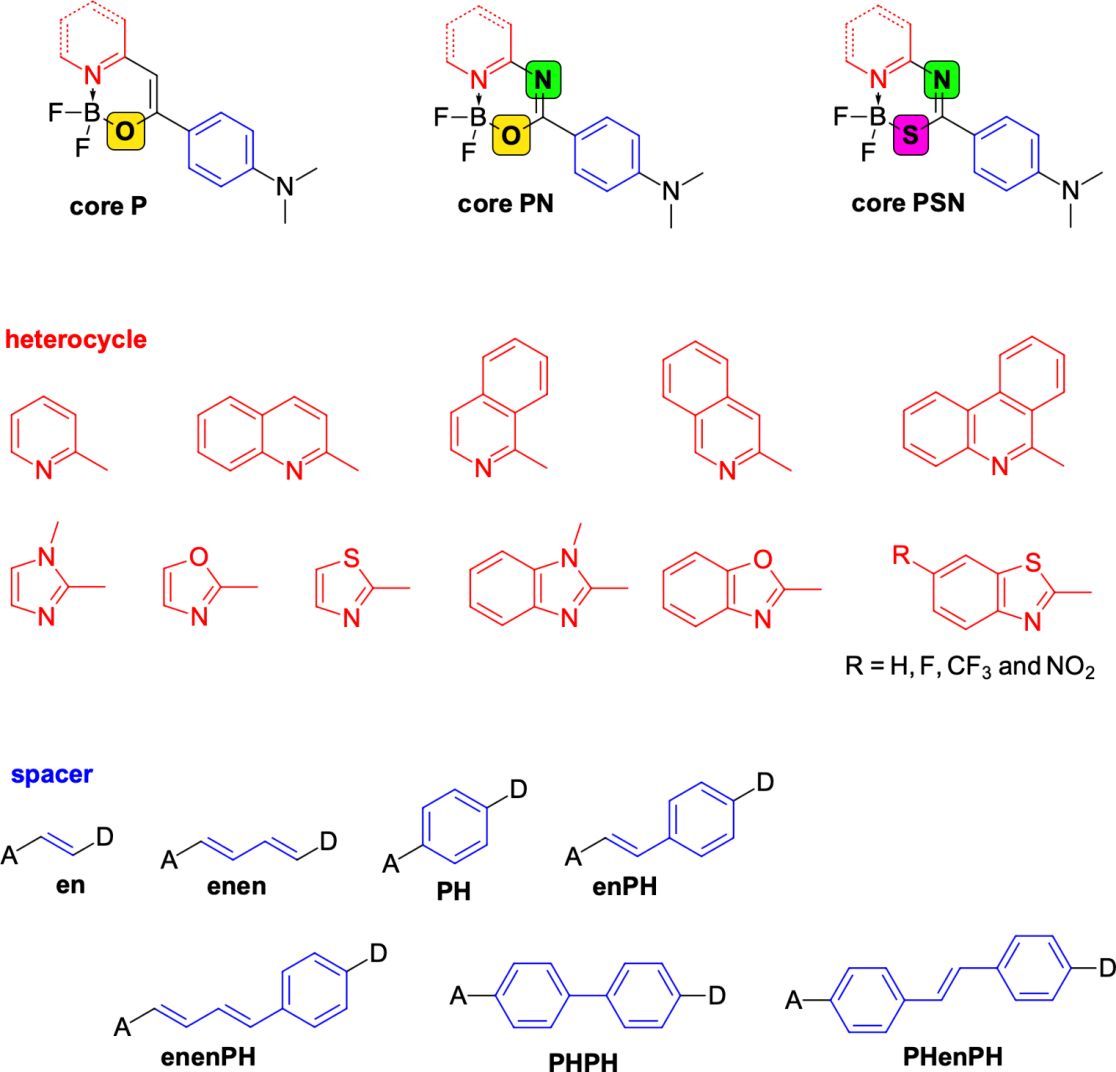
Molecules Studied in the Present Work

In order to study relationships between two- and three-photon absorption, in the present work, we employ electronic-structure theories. It should be emphasized that the two-photon absorption phenomenon was predicted on a purely theoretical basis as early as in 1931 by Maria Göppert-Mayer,^52^ and since then, theory/simulations have been frequently used in studies on multiphoton absorption.^53^ Experimental interpretations of features in 2PA spectra have been supported by theoretical simulations since the 1970s.^54–56^ Thanks to the developments of theoretical models, one may better understand the nonlinear absorption activity.^57–59^ These efforts have been paralleled by the development of methods allowing for efficient simulations of electronic multiphoton absorption properties.^60–68^ Our focus in this work will be on purely molecular parameters defining two- and three-photon transition properties; i.e., we will not attempt to estimate experimental multiphoton absorption cross sections. However, let us highlight that it follows from our previous experimental studies that the majority of molecules studied herein exhibit the values of multiphoton absorption cross sections required for applications, e.g., in bioimaging.^48,69^

The orientationally averaged two-photon transition strength for linear polarization of light δ_2PA_ is defined as follows:^70^

$$\delta_{\mathrm{2PA}}=2\underset{\alpha ,\beta }{\sum }S_{\alpha \alpha }S_{\beta \beta }^{*}+4\underset{\alpha ,\beta }{\sum }S_{\alpha \beta }S_{\alpha \beta }^{*}$$ 1

The evaluation of δ_2PA_ requires the two-photon transition matrix elements *S*_αβ_, which can be derived within the framework of response theory by examining the single residue of quadratic response function:^60^

$$\underset{\omega_{1}\to -\omega_{f}}{\lim}\left(\omega_{2}-\omega_{f}\right){\left\langle \left\langle \mu_{\alpha };\mu_{\beta },\mu_{\gamma }\right\rangle \right\rangle }_{-\omega_{1},\omega_{2}}=S_{\alpha \beta }\left\langle f\right|\mu_{\gamma }\left|0\right\rangle$$ 2

The two-photon transition matrix elements *S*_αβ_ can be expressed explicitly as

$$S_{\alpha \beta }=\underset{i}{\sum }\left(\frac{\left\langle 0\right|\mu_{\alpha }\left|i\right\rangle \left\langle i\right|\mu_{\beta }\left|f\right\rangle }{\omega_{i}-\frac{\omega_{f}}{2}}+\frac{\left\langle 0\right|\mu_{\beta }\left|i\right\rangle \left\langle i\right|\mu_{\alpha }\left|f\right\rangle }{\omega_{i}-\frac{\omega_{f}}{2}}\right)$$ 3

Likewise, orientationally averaged three-photon transition strength for linearly polarized light is given by^71^

$$\delta_{3\mathrm{PA}}=\frac{1}{35}\underset{\alpha ,\beta ,\gamma }{\sum }\left(2T_{\alpha \alpha \beta }T_{\gamma \gamma \beta }^{*}+3T_{\alpha \beta \gamma }T_{\alpha \beta \gamma }^{*}\right)$$ 4

The three-photon transition tensor *T*_αβγ_ can be obtained from the single residue of the cubic response function ⟨⟨μ_α_; μ_β_, μ_γ_, μ_δ_⟩⟩_ω_1_,ω_2_,ω_3__ as

$$\underset{\omega_{3}\to \omega_{f}}{\lim}\left(\omega_{3}-\omega_{f}\right){\left\langle \left\langle \mu_{\alpha };\mu_{\beta },\mu_{\gamma },\mu_{\delta }\right\rangle \right\rangle }_{\omega_{1},\omega_{2},\omega_{3}}=T_{\alpha \beta \gamma }\left\langle f\right|\mu_{\delta }\left|0\right\rangle$$ 5

from which the three-photon transition matrix elements *T*_αβγ_ can be identified as

$$T_{\alpha \beta \gamma }=\underset{P_{\alpha ,\beta ,\gamma }}{\sum }\underset{i,j}{\sum }\frac{\left\langle 0\right|\mu_{\alpha }\left|i\right\rangle \left\langle i\right|\mu_{\beta }\left|j\right\rangle \left\langle j\right|\mu_{\gamma }\left|f\right\rangle }{\left(\omega_{i}-\frac{2\omega_{f}}{3}\right)\left(\omega_{j}-\frac{\omega_{f}}{3}\right)}$$ 6

In order to contribute to the development of “structure-property” relationships, we start with the selection of density functional approximations. The calculations of three-photon transition strengths using coupled-cluster theory are unfeasible for the molecule studies herein. However, given the efficient implementation of the quadratic response theory combined with the RI-CC2 model, it is feasible to select the density functional approximation by computations of two-photon transition strengths. It has been observed that density functional theory provides qualitatively valuable results for purely electronic 2PA with a good balance between accuracy and computational cost.^59,72–79^ More specifically, we used the CAM-B3LYP functional^80^ as it follows from earlier works that it is capable of reliable predictions of two-photon transition strengths for donor–acceptor molecules.^77^ Even though this functional severely underestimates this property in comparison with coupled-cluster theory, the correlation coefficients with reference values are very high and may reach 100% of the variance in the coupled-cluster reference. All molecular geometries for compounds shown in Scheme 1 were optimized using density functional theory with the B3LYP functional^81^ and the 6–31+G­(d) basis set.^82^ Subsequently, (non)­linear optical properties were computed at the RI-CC2/aug-cc-pVDZ and CAM-B3LYP/aug-cc-pVDZ levels of theory^62,83^ using TURBOMOLE^84^ and VeloxChem^85^ quantum chemistry packages, respectively. In the case of the VeloxChem program, the linear, quadratic, and cubic response theory was employed to obtain higher-order transition properties. In this Letter, we consider only *S*_0_ → *S*_1_ transition and we only selected the molecules where corresponding oscillator strength computed using CAM-B3LYP was larger than 0.5 for this transition. This resulted in a set composed of 450 molecules. The summary of RI-CC2 and CAM-B3LYP calculations of two-photon transition strengths is shown in Figure 1. Indeed, it follows from this figure that CAM-B3LYP delivers very good correlation of determination with respect to RI-CC2 reference results (*R* ^2^ = 0.965). This is remarkable agreement given the topological differences in the linkers, cores, and substituents in the acceptor part. The underestimation of two-photon transition strength roughly by a factor of 3 is also in line with other works.^77^ As shown by some of the present authors, these differences can be linked with underestimated excited-state dipole moment in the intramolecular charge-transfer state.^59^ Let us note that the molecular set studied herein is roughly 10 times larger than the previous set of push–pull molecules used for benchmarking density functional approximation.^77,86^ We hope that the reference RI-CC2 data obtained in this study might be useful for the assessment of more approximate methods, e.g., density functional approximations.

**Figure 1:**
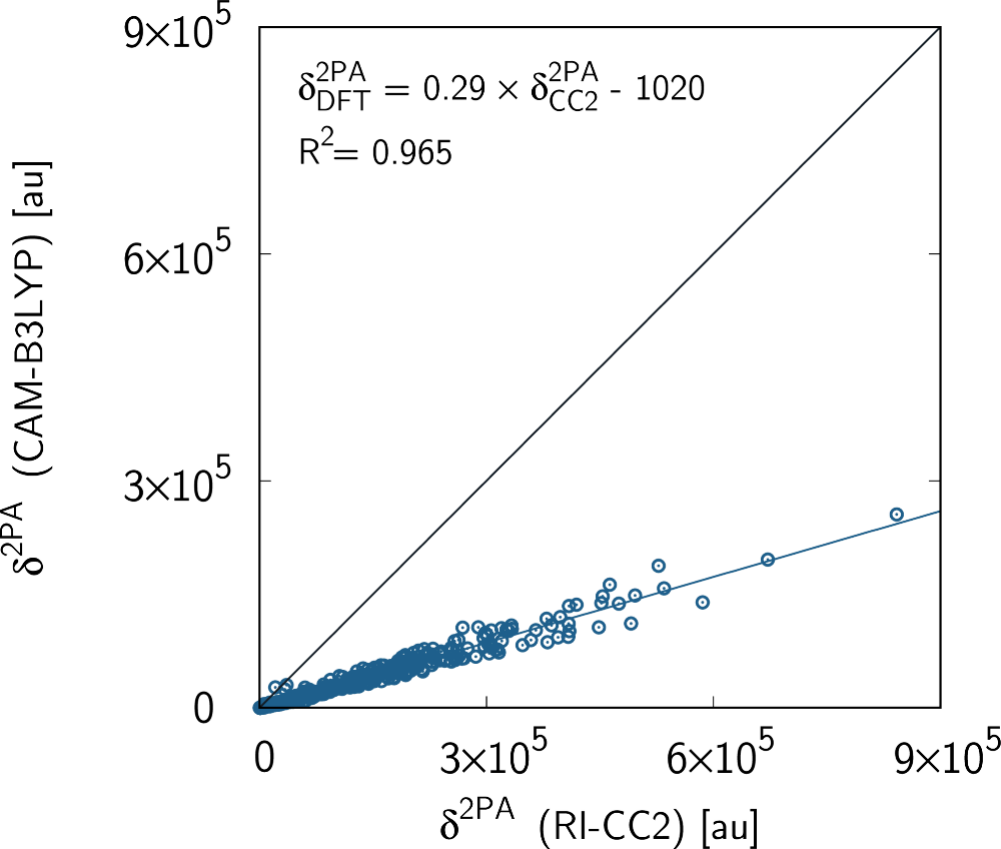
Two-photon *S*_0_ → *S*_1_ transition strengths (given in au) for the set of 450 molecules in the gas phase. The calculations were performed using coupled-cluster method (RI-CC2) and density functional theory (CAM-B3LYP) with the aug-cc-pVDZ basis set.

Given the reliable chemical rankings obtained using the CAM-B3LYP functional, we calculated three-photon transition strengths for the set of 450 molecules. The summary of these calculations is given in Figure 2. The 2PA and 3PA transition strengths show large variations that span several orders of magnitude, i.e., ∼10–10^5^ au (2PA) and ∼10^6^–10^10^ au (3PA). Despite this large spread of values in multiphoton transition strength, there is a clear trend and the corresponding coefficient of determination is *R* ^2^ = 0.862. Note that, in the case of values of 2P transition strengths larger than 10^5^ au, the deviation from the linear model is clearly larger. This can be traced back to the significant chemical variability of cores (different heterocyclic moieties fused) and linkers. In order to shed light on this aspect, we analyzed separately three different linkers, i.e., butadienylene-phenylene (**enenPH**), biphenylene (**PHPH**), and stilbenediyl (**PHenPH**). The results of this analysis are presented in Figure 3. As seen, for the latter two linkers, the coefficient of determination is much larger than for the whole set (0.948 for **PHPH** and 0.955 for **PHenPH**). Let us now shed light on the dependence of 2PA and 3PA properties on structural motifs present in the set. Among the 450 molecules studied, the largest multiphoton transition strengths are found for nitrobenzothiazole combined with a core denoted as **PSN** and a linker, **PHenPH**. The corresponding values of two- and three-photon transition strengths are as large as 2.6 × 10^5^ and 5.0 × 10^10^ au. The compounds containing stilbenediyl as a spacer exhibit the best 2PA and 3PA transition strengths, regardless of the heterocycle or core employed. On the other hand, smaller spacers such as ethene (**en**) or butadiene (**enen**) are associated with a considerable decrease in 2PA and 3PA properties. The core structure between the spacer and the electron acceptor varied among the molecules. Each core consisted of a six-membered coordination ring containing a BF_2_ group, along with either one heteroatom (O: **P** core) or two heteroatoms (N, O: **PN** core; N, S: **PSN** core). The introduction of an additional heteroatom led to a significant improvement in 2PA and 3PA transition properties, observed for the **PSN** core, compared to the **P** and **PN** cores. Compounds containing single-ring acceptors, such as *N*-methylimidazole, oxazole, or thiazole, consistently exhibited the lowest 2PA and 3PA strength values, irrespective of the linker or core. Significant improvements in these properties were observed when an additional fused benzene ring was incorporated into the acceptor structure (e.g., benzimidazole, benzoxazole, and benzothiazole). For 2PA, the enhancements ranged from approximately 30% (on passing from thiazole to benzotiazole) to 50% (on passing from imidazole to benzimidazole), while for 3PA the increase was even more pronounced from about 50% (thiazole to benzothiazole) to 90% (imidazole to benzimidazole). A comparable enhancement was achieved by replacing pyridine with quinoline. Subsequently, we examined the influence of substituents on the heterocyclic acceptor with benzothiazole serving as a model system. A clear improvement in the properties of both 2PA and 3PA strengths was observed when electron-withdrawing groups such as F, CF_3_, or NO_2_ were employed. Among these, nitro substitution had the strongest impact, delivering more than a 2-fold increase in 2PA strength and over a 3-fold increase in 3PA strength across all tested cores and **PHenPH** spacer.

**Figure 2:**
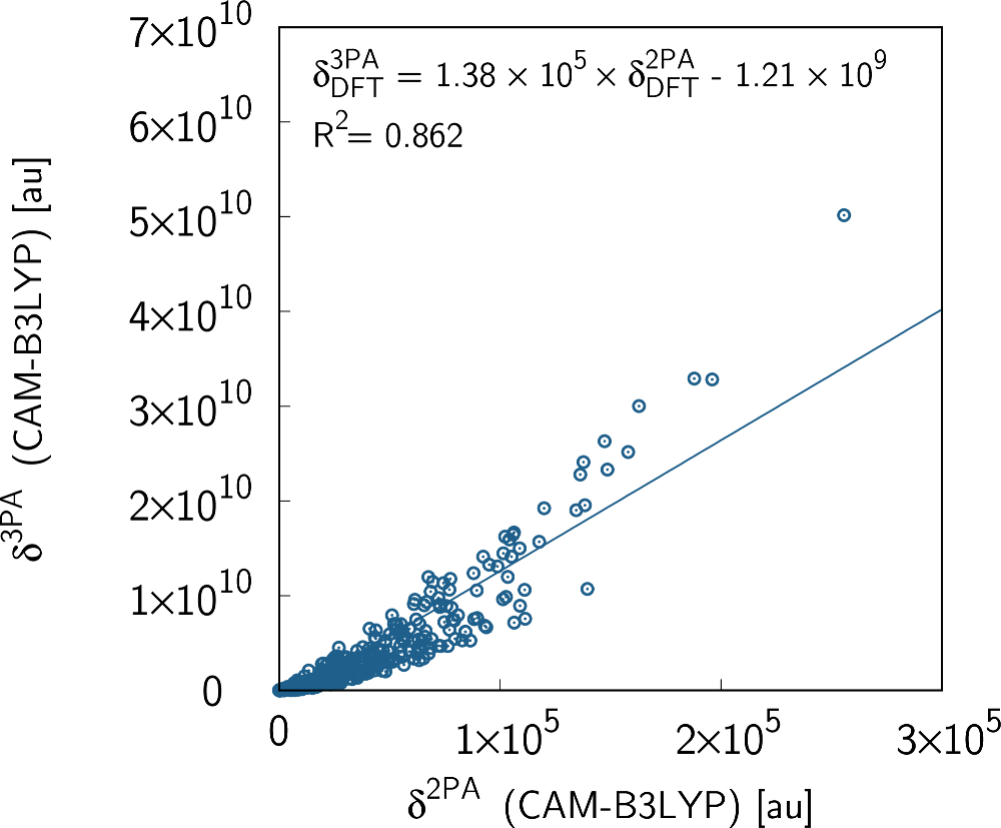
Two- and three-photon *S*_0_ → *S*_1_ transition strengths (given in au) for the set of 450 molecules in the gas phase. The calculations were performed using the CAM-B3LYP functional with the aug-cc-pVDZ basis set.

**Figure 3:**
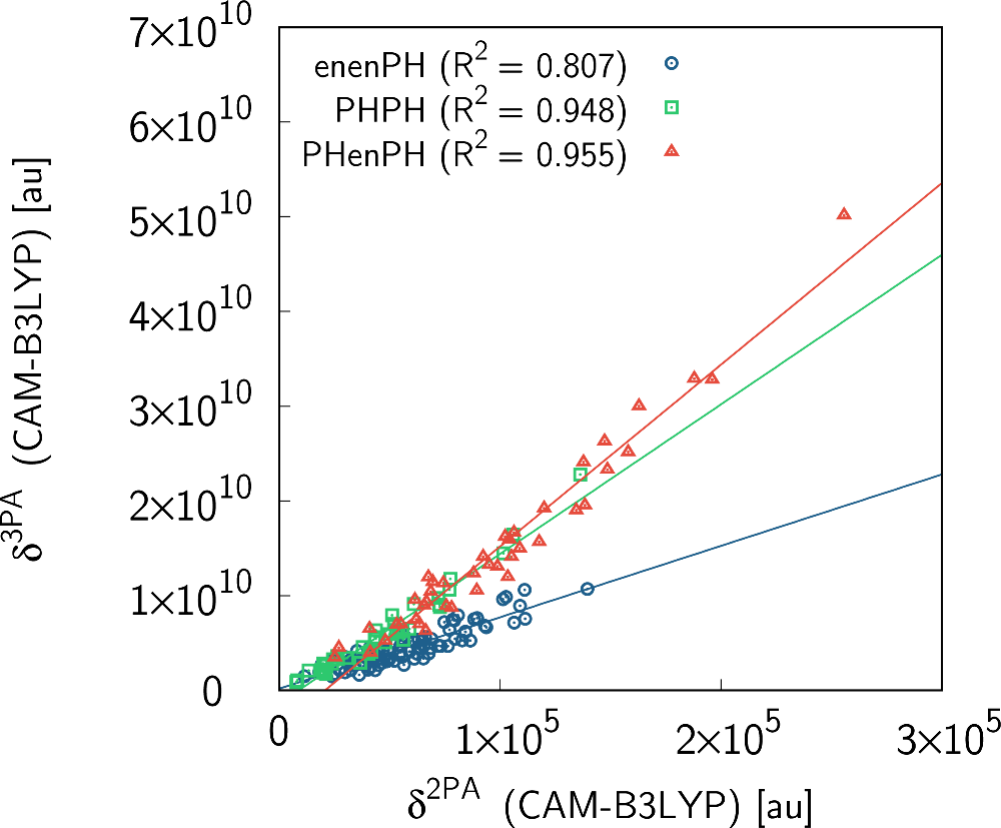
Two- and three-photon *S*_0_ → *S*_1_ transition strengths (given in au) for the subset of molecules containing enenPH, PHPH, and PHenPH linkers. The calculations were performed for molecules in the gas phase using the CAM-B3LYP functional with the aug-cc-pVDZ basis set.

Since the whole series contains 450 structures, we will now consider a subset of the most interesting ones. Sorting the structures by these two properties with a threshold for 2PA higher than 10^5^ au and 3PA higher than 1.4 × 10^10^ au, simultaneously, forms a set composed of 20 molecules (less than 5% of the whole set). Within this group, 17 molecules carry stilbenediyl as a spacer joining acceptor and donor, while only 3 carry biphenylene. At the same time, 12 molecules from the group carry a sulfur atom in their structure. The correlation between 2PA and 3PA strengths in the series is high and equals 0.963. For molecules that are in the subgroup of structures characterized by 2PA strength equal to 1.0 × 10^5^ au or higher, there are 18 of 28 structures that carry sulfur in the acceptor part (**PSN** core). The same number of dyes have stilbenediyls in their structure. On the other hand, in the case of threshold of 3PA strength equal to 10^10^ au or higher, there are 18 out of 34 molecules that carry sulfur and 27 of 34 carry stilbenediyl as spacer. The above-mentioned numbers suggest that, in order to maximize 2PA transition strength, the presence of the sulfur bonded with boron is equally important as the use of the longest spacer (18/28 = 0.64, meaning 64% of molecules from the group of 28 selected). On the other hand, in the case of 3PA absorption, one observes that the longest spacer has a higher influence on maximization of the cross-section (27/34 = 0.79) than the presence of a sulfur–boron moiety (18/34 = 0.53). Finally, in order to maximize both 2PA and 3PA strengths, the long spacer is more potent (17/20 = 0.85) than the presence of the sulfur atom (12/20 = 0.60) among the tested structures. This observation might be applied in the design and syntheses of multiphoton absorbers. Moreover, it is necessary to mention that the use of stilbenediyl in CT dyes may introduce a risk of trans/cis photoisomerizarion, which may lead to photobleaching.^87,88^

In summary, we studied “structure-property” relationships for two- and three-photon absorption using computational quantum chemistry methods. We presented the results of the analysis for a set comprising 450 organoboron dyes representing various core topologies combined with a palette of linkers. The combination of these structural moieties leads to donor–acceptor architectures manifested by the presence of a low-lying electronic excited state of intramolecular charge-transfer character. The multiphoton excitation to this state is intense and significant from the application point of view and, therefore, became our focus for the analysis of multiphoton properties. The analysis carried out in this work clearly demonstrates that there is strong correlation between the intensities of the two- and three-photon transition to the lowest intramolecular charge-transfer state; i.e., the coefficient of determination between 2PA and 3PA strengths for the whole set is *R* ^2^ = 0.862. However, the corresponding values of *R* ^2^ for a palette of heterocycles/cores combined with the most efficient linkers are much higher, i.e., 0.948 (**PHPH**) and 0.955 (**PHenPH**). These high values of the coefficient of determination deliver strong evidence that the developed design rules aiming at maximizing two-photon absorption efficiency will also be useful in designing three-photon absorbers. This conclusion thus holds for donor–acceptor systems, and more systematic studies are needed for other molecular topologies. These calculations were performed using the CAM-B3LYP functional selected based on the comparison with the coupled-cluster RI-CC2 model; i.e., we found that the coefficient of determination between 2PA strengths computed using CAM-B3LYP functional and RI-CC2 reference is as large as *R* ^2^ = 0.965, indicating the reliability of this functional for predicting chemical rankings of multiphoton absorbers.

## Supplementary Material





## Notes

Cover page for ZIP file (PDF)
Atomic coordinates of 450 molecules (ZIP)
