# Supplementary material for: Structure of Organoboron Dyes and Multiphoton Absorption: Insights from Theory
Source: J Phys Chem Lett. 2025 Sep 9;16(37):9693–700. doi: 10.1021/acs.jpclett.5c02066 (PMC12451736; doi:10.1021/acs.jpclett.5c02066)
Supplement: Supplementary file 1 [file jz5c02066_si_001.pdf]

Supporting Information: Structure of Organoboron Dyes and Multiphoton Absorption: Insights from Theory

Karan Ahmadzadeh,[*,†] Natasza Trzęsowska,[‡] Rafał Wysokiński,[‡] Zilvinas Rinkevicius,[¶] Robert Zaleśny,[‡] Wei Hu,[†] Borys Ośmiałowski,[*,§] and Hans Ågren[*,‡]

†*Hefei National Research Center for Physical Sciences at the Microscale, University of Science and Technology of China, Hefei, Anhui 230026, China*

‡*Faculty of Chemistry, Wrocław University of Science and Technology, Wyb. Wyspiańskiego 27, PL–50370 Wrocław, Poland*

¶*Division of Theoretical Chemistry and Biology, School of Engineering Sciences in Chemistry, Biotechnology and Health, KTH Royal Institute of Technology, SE-100 44 Stockholm, Sweden*

§*Faculty of Chemistry, Nicolaus Copernicus University, Gagarina Street 7, Toruń, PL-87-100, Poland*

E-mail: karan@ustc.edu.cn; borys.osmialowski@umk.pl; hans.agren@pwr.edu.pl

---

The accompanying ZIP archive file contains the geometries of molecules studied in the manuscript (in Ångstrom units).
